# Supplementary material for: Physical reality of the Preisach model for organic ferroelectrics
Source: Nat Commun. 2018 Oct 23;9:4409. doi: 10.1038/s41467-018-06717-w (PMC6199281; doi:10.1038/s41467-018-06717-w)
Supplement: Supplementary file 1 — Supplementary Information [file 41467_2018_6717_MOESM1_ESM.pdf]

Supplementary Information

Physical reality of the Preisach model for organic ferroelectrics

Urbanavičiūtė *et al.*

Supplementary Figures

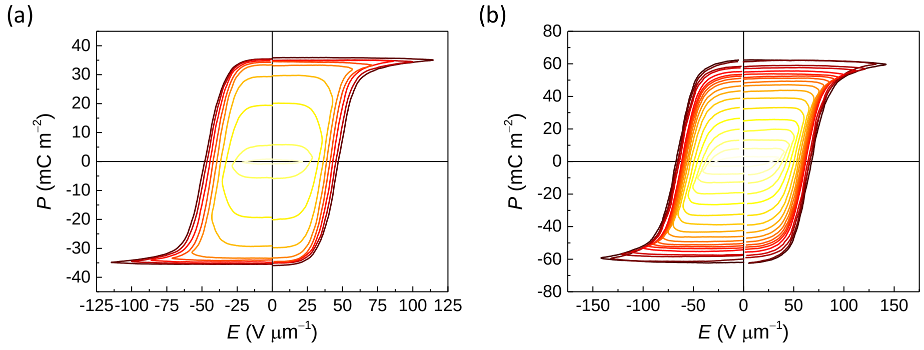

Supplementary Figure 1. Experimental *P-E* characteristics with minor loops. (a) BTA-C10 (55 °C) and (b) P(VDF-TrFE) (23 °C) devices at 10 Hz applied field frequency. Measurement performed using the double-wave method (DWM).

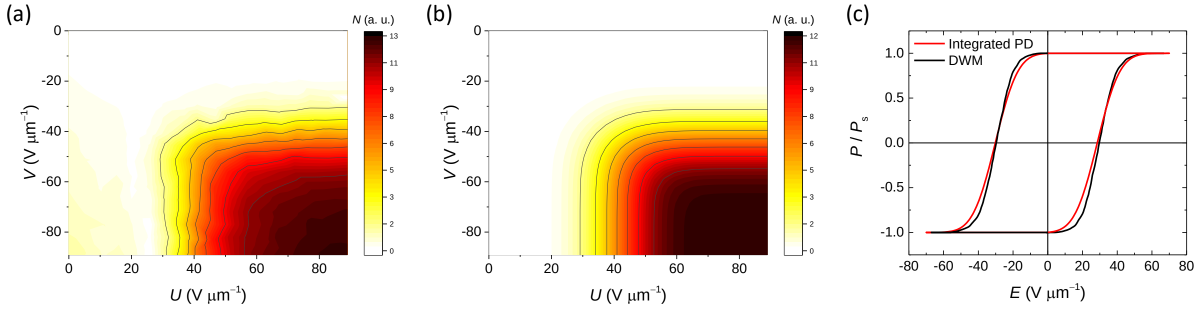

Supplementary Figure 2. Demonstrating the validity of the PD measurement and fitting procedure. (a) The cumulative PD for BTA from which Fig. 3(a) is derived, (b) is the corresponding fit with an integrated Gaussian with an R^2 of 0.99386. (c) Closely matching *P-E* curves for BTA as measured by the DWM (major loop, black line) and as obtained from integrating the whole PD in panel (a) (red line).

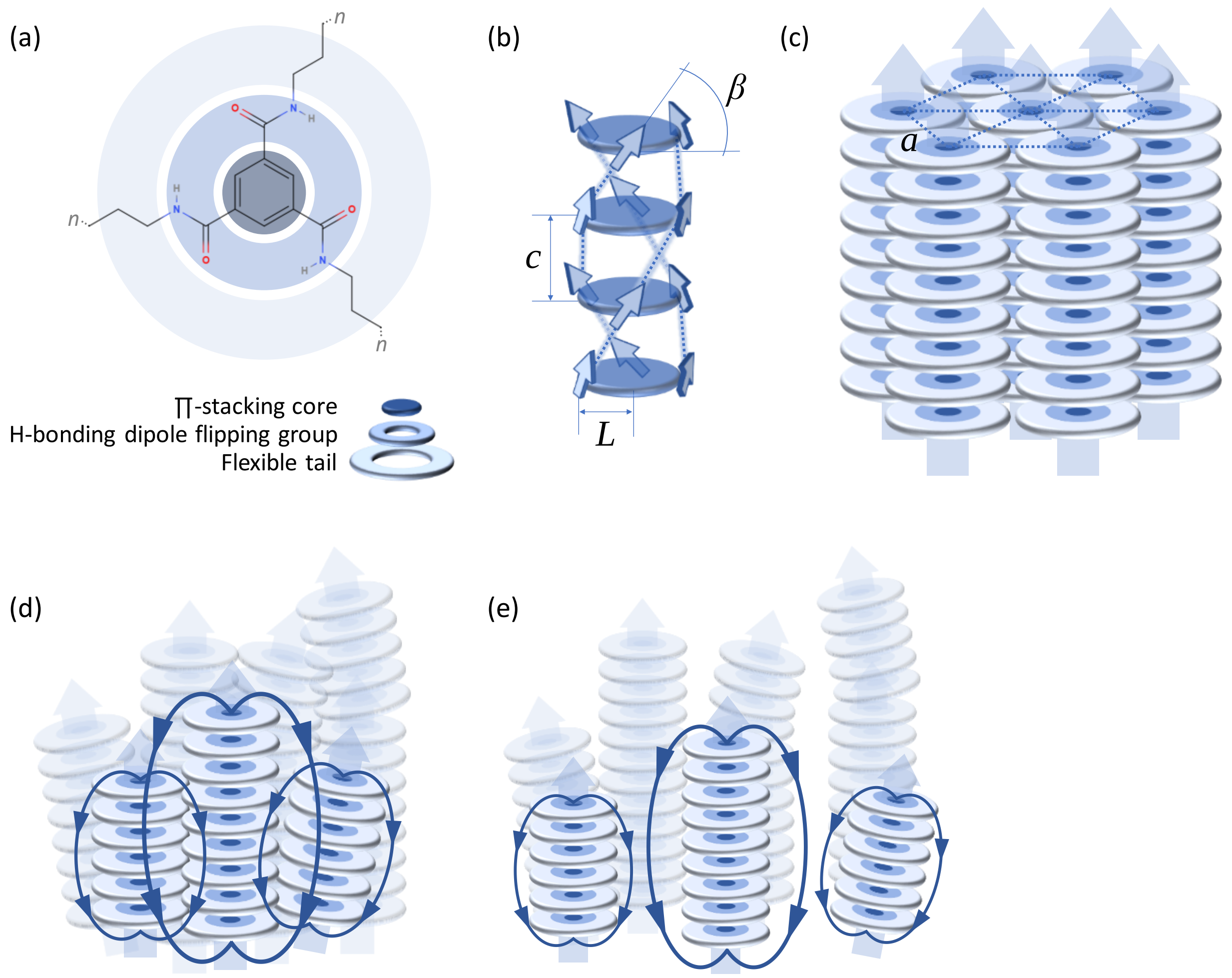

Supplementary Figure 3. Overview of the morphology of BTA as used in the model. The BTA molecule (a) stacks in columns, forming helical hydrogen bonds (b). Columns pack in a hexagonal lattice (c). Disorder is introduced using the clusters shown in (d,e). Variation in the cluster size and the strength of inter-cluster interactions determine the form of the Preisach distribution.

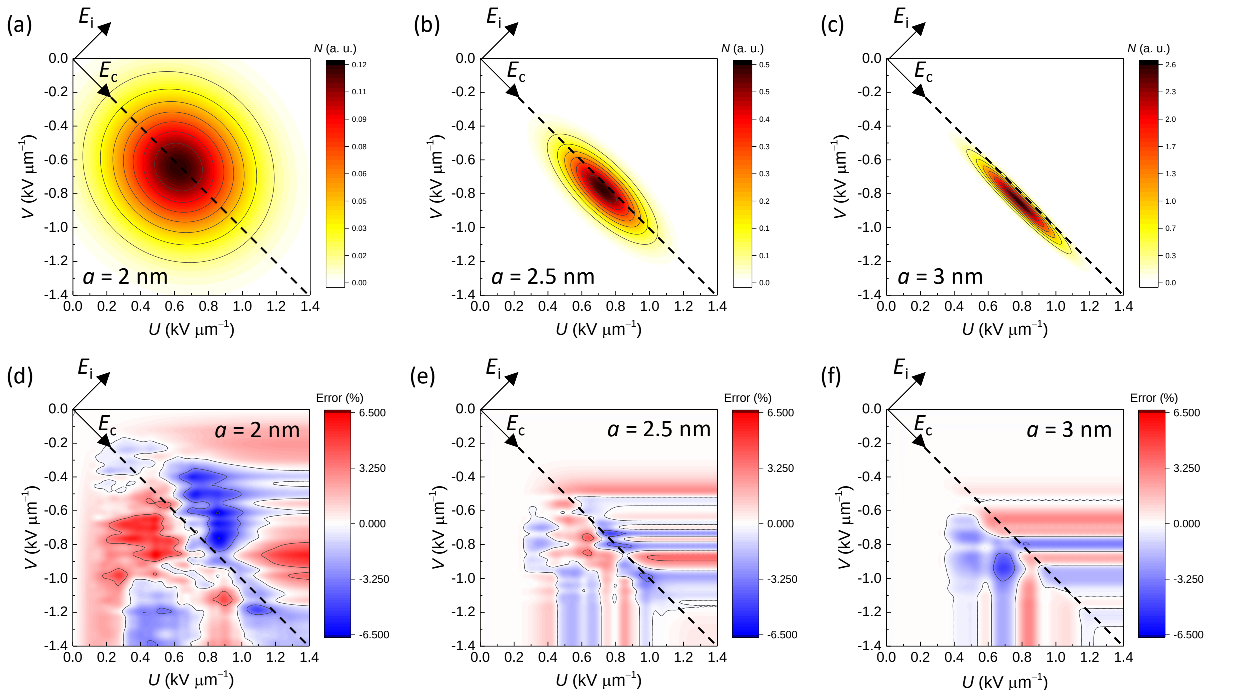

Supplementary Figure 4. Fits and errors of the simulated Preisach distribution. (a–c) Preisach distributions extracted from simulated polarization switching for BTA with cluster separation a of 2, 2.5 and 3 nm, showing transition from BTA- to P(VDF-TrFE)–like Preisach distribution. Panels (a,c) are included in the main text as Fig. 4(a,b). The rotated coordinate system is shown and the dashed line indicates $U = -V$. (d–f) The percentage error between the simulated integrated PD and the double error function fit, for the indicated cluster separation a, corresponding to panels (a–c). The color-scale is the same in all plots. Panels (d,f) correspond to panels (a,b) in Figure 4 of the main text.

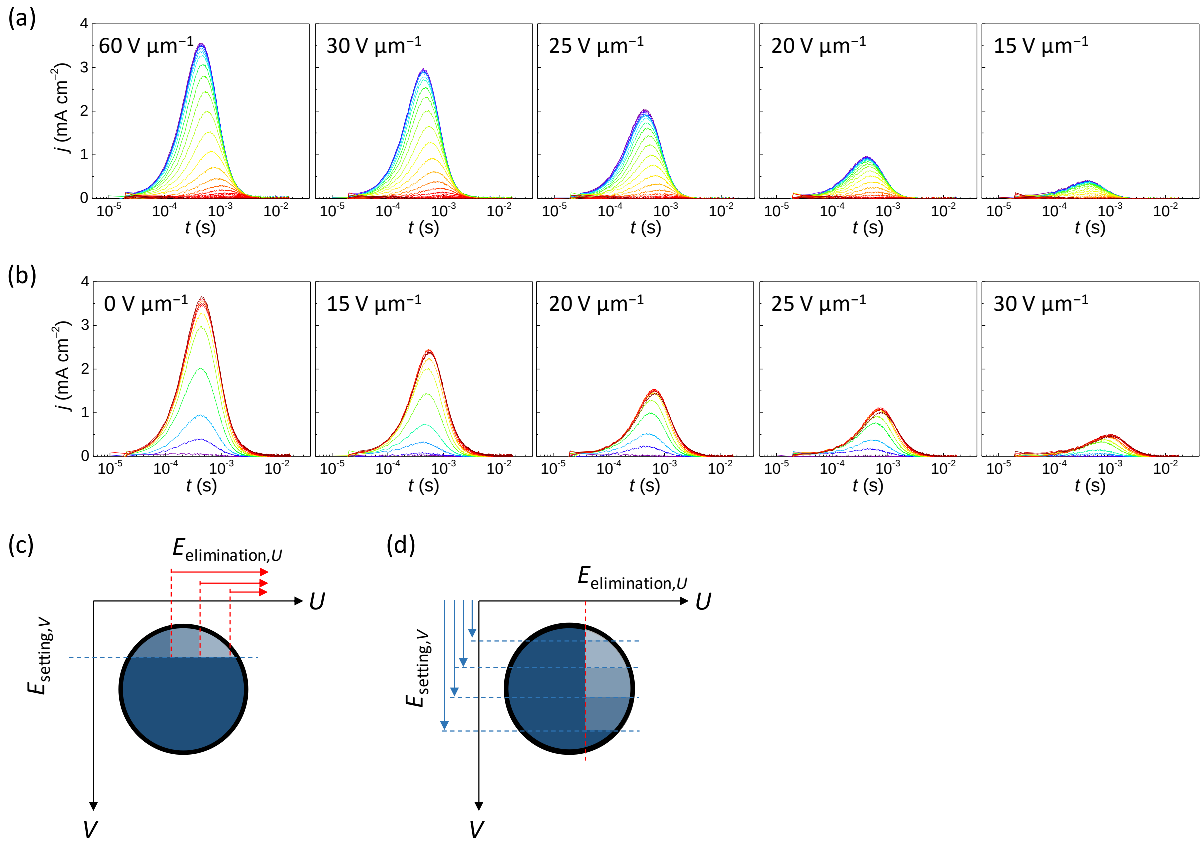

Supplementary Figure 5. Examples of transient switching currents of the partially filled PD. The measurement method is described in Fig. 5 of the main text. (a) Changing the elimination field $E_{set,U}$ (increasing from blue to red), while the setting field $E_{set,V}$ is kept constant at the value given in the legend. The probed PD part is schematically illustrated in panel (c). (b) Changing the setting field $E_{set,V}$ (increasing from blue to red) while the elimination field $E_{set,U}$ is set at the value given in the legend, is schematically illustrated in panel (d). The time when the switching transient current reaches a maximum is used as the switching time to construct Fig. 6(a,b).

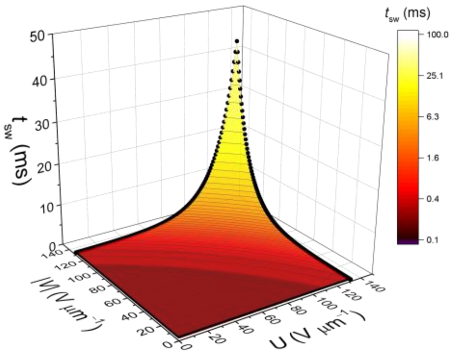

Supplementary Figure 6. Alternative data for fitting to the bivariate Eq. 3b. Fitting is performed using only experimental data for projections of the actual dependence (black circles). An example of BTA.

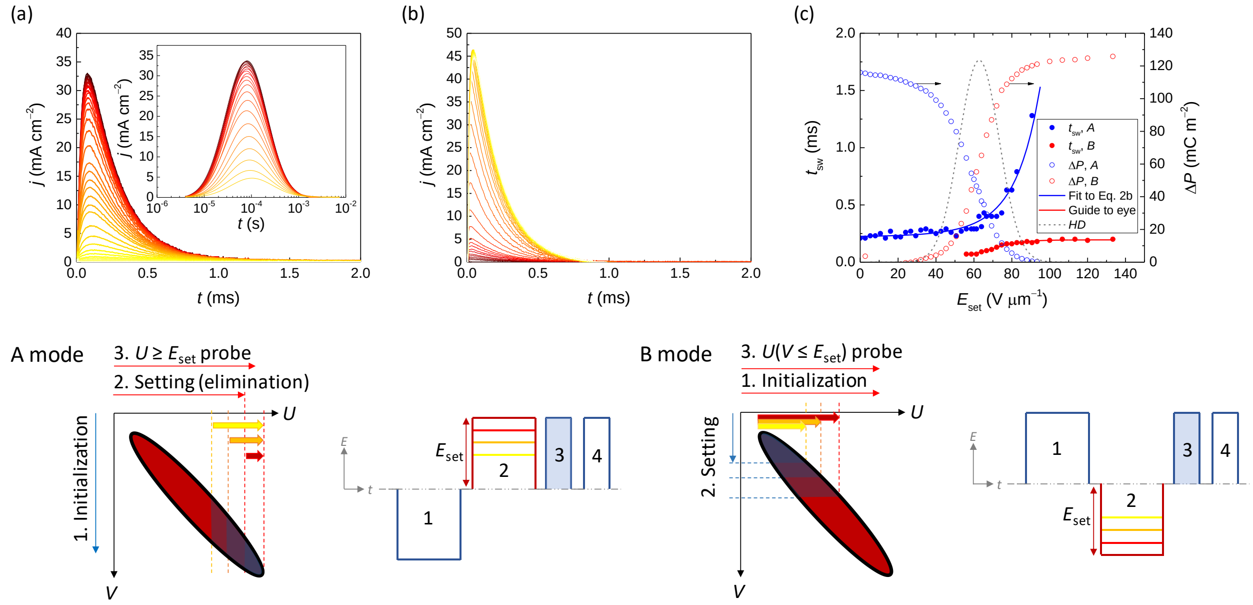

Supplementary Figure 7. Partial PD switching kinetics obtained for a P(VDF-TrFE) device. Panels (a) and (b) correspond to the measurements *A* and *B* as explained in the main text, respectively. (c) The extracted switching time (full symbols) and switched polarization part (open symbols) dependence on the setting field in scenario *A* (blue) and *B* (red). Schematics below are to illustrate the scenario *A* and *B* in context of the elliptical Preisach distribution. Thick arrows indicate the measured PD part.

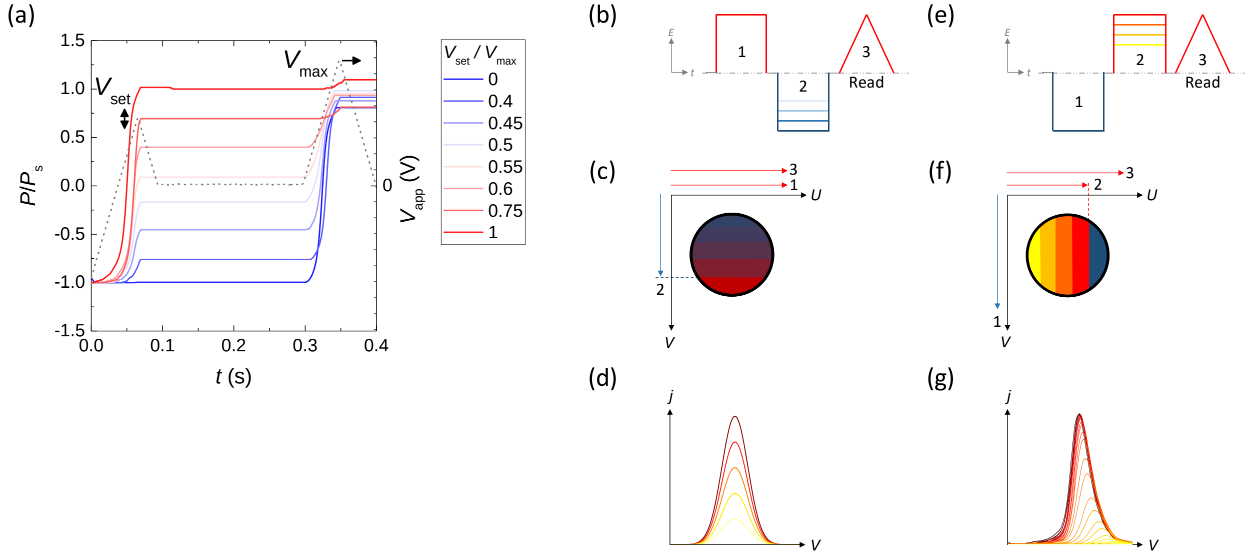

Supplementary Figure 8. 3-bit memory (8 states) in BTA. (a) Experimental demonstration of the 3-bit memory in BTA at room temperature. The intermediate states are set by varying the amplitude of the setting pulse V_{set} as indicated. The slight differences between the fully polarized states are due to non-

switching background currents. Two implementation modes (b–d) and (e–g) are possible and would give similar results in terms of switched polarization. (b,e) is the setting pulse sequence, which is used to select a particular part of the Preisach distribution as shown in (c,f). The current density response measured at step 3 is given in (d,g).

Supplementary Tables

Supplementary Table 1. Default parameters as used in the interacting dipole model (see also Supplementary Figure 3).

	Parameter	Value	Unit
**Morphology**	Helical pitch	6	molecules
	OOP rotation β	40	degrees
	Intercolumnar distance a	1.67	nm
	Inter-disc distance c	0.35	nm
	Dipole distance from center *L*	0.28	nm
**Disorder**	Cluster size	7	molecules
	Cluster size disorder (Parabolic width)	2	molecules
	Cluster Positional disorder (Parabolic width)	0.02	nm
**Simulation**	Box size in the *x* direction	10	molecules
	Box size in the *y* direction	10	molecules
	Box size in the *z* direction (column length)	60	molecules
	Interaction cut-off range	40	dipoles
**Material**	Permanent dipole moment μ	4	D
	Polarizability α	1	$e\text{Å}^2 V^{-1}$
	Effective permittivity ϵ_r	2	-

Supplementary Table 2. Fitting parameters for the fits of the switching kinetics in Figures 6(a) and 7. The top three rows are free parameters; the bottom three rows are given by the experimental conditions. Two slightly different sets of parameters are found for BTA due to slight differences in experimental conditions.

	**BTA Figure 6(a)**	**BTA Figure 7(a,b)**	**P(VDF-TrFE) Figure 7(c,d)**	**Units**
w_b	0.1	0.11	0.17	eV nm^{-3}
ν_0	0.16	1	0.44	THz
t_0	0.39	0.49	0.03	ms
d	NA	2	2	dimensionless
P_r	0.035	0.05	0.1	mC m^{-2}
t	0.05	1	1	s
E_{app}	60	40	80	MV m^{-1}

Supplementary Notes

Supplementary Note 1: Details on the Preisach distribution electrostatic model

In the model we reduce the complex hierarchical morphology of BTA molecules to a collection of point dipoles and their interactions. The morphology of the system is shown in Supplementary Figure 3. Each BTA molecule contains three dipoles that tilt out of plane to form a helical structure with the rest of the column, resulting in a macro-dipole. Columns are disordered and contain defects that separate perfectly ordered clusters. For simplicity, we assume that the individual dipole flipping process within a cluster can be ignored and only consider the flipping of clusters as a whole. Furthermore, periodic boundary conditions are used. An overview of typical simulation parameters is given in Supplementary Table 1.

For each dipole *i* the interactions with neighboring dipoles are calculated up to a certain cut-off range r_c. Including the applied electric field E_{applied}, the energy of a dipole is then

$$U_i = -\overrightarrow{\mu_i} \cdot \left(\overrightarrow{E_{\mathrm{applied}}} + \sum_{r_{ij}<r_c} \frac{1}{4\pi\epsilon_r\epsilon_0 r_{ij}^3}\left[\overrightarrow{\mu_j} - 3\widehat{r_{ij}}\left(\overrightarrow{\mu_j}\cdot\widehat{r_{ij}}\right)\right]\right), \quad (1)$$

with μ the dipole moment, r_{ij} the distance vector between dipole *i* and *j*, and $\epsilon_r\epsilon_0$ the permittivity of the material.

On top of the permanent dipoles of the amide groups, we should also consider induced dipoles. Unlike the permanent dipoles, these induced dipoles are not restricted to only two fixed orientations, as they will lie along the direction of the local field $\overrightarrow{E_{\mathrm{loc}}}$:

$$\overrightarrow{\mu_{\mathrm{tot}}} = \overrightarrow{\mu_{\mathrm{perm}}} + \overrightarrow{\mu_{\mathrm{ind}}} = \overrightarrow{\mu_{\mathrm{perm}}} + \alpha * \overrightarrow{E_{\mathrm{loc}}}, \quad (2)$$

with α the electronic polarizability.

The Preisach distribution is now simulated by replicating the experiment and sweeping the electric field step by step. At each step in a simulation, all interactions are recalculated, and for each cluster it is determined if it is energetically favorable to flip. At each step, the number of flipped dipoles is registered, which will finally give the integrated PD. This integrated distribution is fitted in the same way as the experimental data. As the model does not comprise kinetic processes and is temperature independent, the simulated fields correspond to intrinsic rather than extrinsic switching and exceed the experimental values by at least an order of magnitude, as predicted by Eq. 2 in the main text with *T* = 0 K.

The error of the fits to all simulated integrated PDs is shown in Supplementary Figure 4. The fit for the narrow distributions (b and c) is decent with the error within a few percent (e,f). However, discrepancies appear for the broader distribution (a and d), indicating that the simulated distribution deviates from the simple double Gaussian form. This means that either our approximation of the PD with a Gaussian is wrong, or the model cannot completely describe the situation for high intercolumnar interactions. The latter is plausible considering the simplicity of the model. On the other hand, it is also to be expected that in reality the distribution is indeed more complex than just a double Gaussian. Experimentally, a Gaussian is found to best fit the data, but the actual measurement noise would hide deviations of the size found in any of the simulations.

Supplementary Methods

Device fabrication and pre-conditioning

Thin film metal-ferroelectric-metal (MFM) capacitor devices of BTA-C10 were formed by spin-coating (500–1000 rpm) of a 40 mg/ml chloroform solution on a chemically cleaned glass substrate with patterned aluminum (or chromium/gold) bottom electrodes. Before thermal vacuum deposition of the aluminum (or gold) top electrodes, spin-coated films were annealed at 60 °C for 15 min to completely evaporate the solvent. The prepared MFM devices were 0.01–1 mm^2 in area. Typical film thickness was 400–700 nm, as measured by a Bruker Dektak XT profilometer.

In the as-cast organic ferroelectric film molecular columns lie in-plane to the electrode. When molecular dipoles are oriented in this way, no polarization can be measured in the bottom-top electrode geometry. Therefore prior to the electrical measurements, the devices are treated by a field-annealing procedure, when at low viscosity conditions (∼100 °C) with the help of an alternating external field molecular bundles are forced to stand perpendicularly to the electrodes (to some scale, determined by the material's tendency towards disorder). The followed field-cooling freezes the system in this quasi-orderly state. Due to π-stacking of the benzene core and hydrogen bonding of the amide groups, accompanied by alkyl chain freezing, the hexagonal packing remains stable even without external field. This has been previously tested by polarized light optical microscopy (POM) and can be seen from unchanged current transients corresponding to polarization reversal after a long waiting time. Therefore only the polarization switching current, rising from the dipole rotation, reflects in the quasi-statically measured *P*-*E* curves (see Electrical characterization), once the device is properly conditioned.

P(VDF-TrFE) capacitor devices were prepared from 60 mg/ml cyclohexanone solution, which was stirred overnight and filtered before spin-coating (2000 rpm) on patterned chromium/gold (5/50 nm) electrodes on chemically cleaned glass substrates. Deposited films were annealed at 140 °C for 2 hours to increase crystallinity. The procedure was repeated until desired thickness was obtained (400–700 nm). Top gold electrodes were subsequently deposited by thermal evaporation in vacuum to form cross-bar structure capacitor devices. Ferroelectric devices were further characterized as described below.

Electrical characterization

The input signal waveform is supplied by a Tektronix AFG3000 Arbitrary Function Generator and is amplified by a TREK PZD350A high voltage amplifier. The device response is visualized by a Tektronix TBS1000B Digital Oscilloscope.

The polarization loops are obtained by integration of the switching current transients. We use a quasi-static mode, better known as the Double Wave Method (DWM), where the non-switching current is subtracted from the initial signal to avoid displacement and leakage (if any) inputs in the *P*-*E* curves.
